# Reply to: Reassessing data quality underlying the recently updated floristic map of the world

**DOI:** 10.1038/s41467-024-47544-6

**Published:** 2024-05-02

**Authors:** Yunpeng Liu, Xiaoting Xu, Dimitar Dimitrov, Carsten Rahbek, Zhiheng Wang

**Affiliations:** 1 Institute of Ecology, College of Urban and Environmental Sciences, and Key Laboratory of Earth Surface Processes of Ministry of Education, Peking University, Beijing, 100871 China; 2 Center for Macroecology, Evolution and Climate, Natural History Museum of Denmark, University of Copenhagen, Universitetsparken 15, DK-2100 Copenhagen Ø, Denmark; 3 Key Laboratory of Bio-Resource and Eco-Environment of Ministry of Education, College of Life Sciences, Sichuan University, Chengdu, 610065 Sichuan China; 4 Department of Natural History, University Museum of Bergen, University of Bergen, Postbox 7800, 5020 Bergen, Norway; 5 Center for Global Mountain Biodiversity, GLOBE Institute, University of Copenhagen, Universitetsparken 15, 2100 Copenhagen, Denmark; 6 Danish Institute for Advanced Study, University of Southern Denmark, 5230 Odense M, Denmark

**Keywords:** Biodiversity, Macroecology

**replying to** H. Qian *Nature Communications* 10.1038/s41467-024-47543-7 (2024)

A Matters Arising article^1^ has raised concerns about the general distribution data used in our study^2^, suggesting the use of non-native distribution records and incomplete sampling, and arguing that these issues may bias our findings. After a careful examination of our data and a re-analysis, we found that these issues and their effects on our results are not as serious as suggested in the Matters Arising. We further updated our databases based on the latest version of the POWO database and repeated the regionalization analyses.

## Cleaning of non-native distributions

The Matters Arising argued that our database contained an extremely high proportion of non-native distributions. Specifically, Dr. Qian concluded that >7500 genera in our database had non-native distribution data, and the total number of non-native distributions ca. 65,000. We conducted a careful evaluation of the non-native distributions contained in our database using the latest version of the Plants of the World Online (POWO) (https://powo.science.kew.org; assessed on Aug 7th, 2023). First, in total, the latest POWO database contains distribution data for 14,020 genera (including not only angiosperms but also other plant clades, such as gymnosperms), among which 2757 genera have non-native distribution data. The overall number of non-native distribution records at the genus level is 49,526. These total numbers of genera with non-native distribution data contained in the POWO database are already much lower than those reported in the Matters Arising.

Second, we compared the non-native distribution data of the POWO database with our database (see Methods for details of the analysis and Supplementary Data 2 in ref. ^2^). Due to the inconsistency between the boundaries of the POWO map and the geographical standard units (GSU) used in our study, we overlaid the two maps to match our GSUs with the POWO geographic units. A GSU is assigned to a POWO unit if >25% of its area is overlapped with a POWO unit. We found that 401 GSUs were matched to one POWO unit and 19 were covered by two POWO units. Then, non-native distributions of genera in our database were identified. We found that only 10.5% of the genera (1331 genera) contained in our database had non-native distributions. The number of the non-native distributions in our database is 16,566, which accounts for 4.3% of the total distributions database (384,133 data entries). To confirm these results, we further increased our threshold of accuracy (see Methods) for matching our GSUs with POWO units and repeated the analysis. We found the results were even more conservative: 6.6% of all genera (836 genera) had 10,624 non-native distributions.

**Fig. 1:**
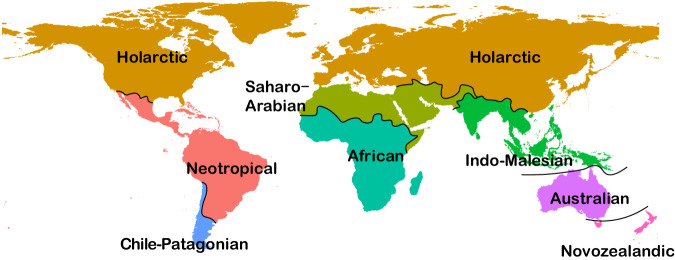
Floristic realms of the world based on all the genera in our study^2^. Introduced data listed in POWO are removed, and we also included the distribution records from POWO that are new to our database and can be precisely adapted to our database.

**Fig. 2:**
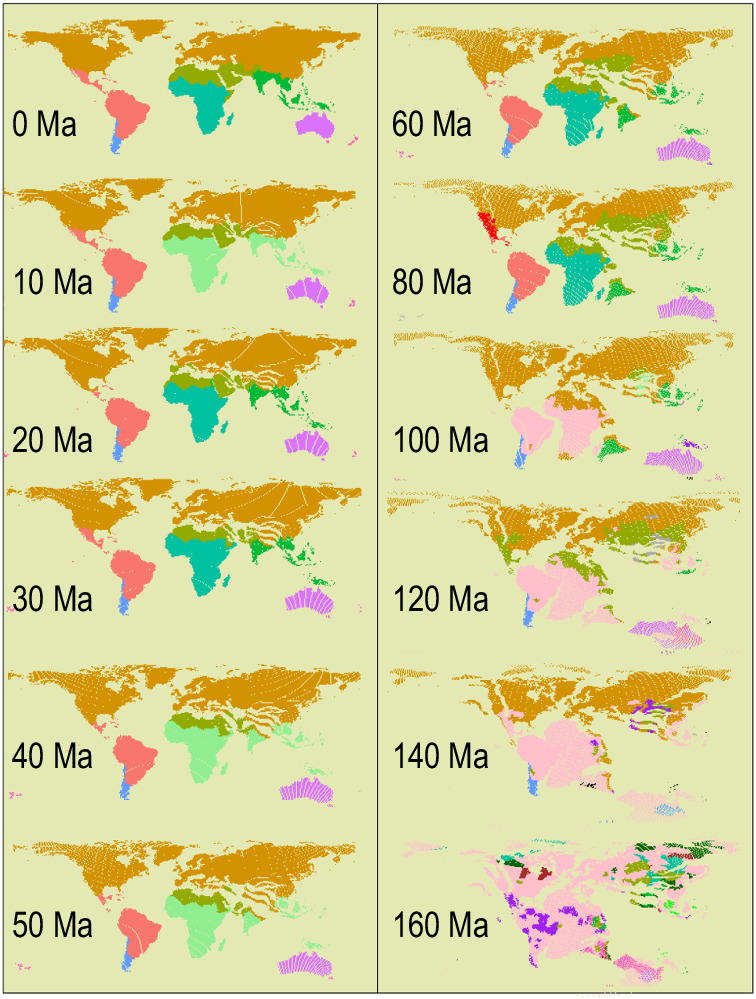
Chronology of the present-day floristic realms. Introduced data listed in POWO are removed, and we also included the distribution records from POWO that are new to our database and can be precisely adapted to our database.

These results clearly indicate that the proportion of non-native distributions in our database is not as substantial as suggested in the Matters Arising. One possible explanation is that the POWO map might not have been correctly matched with our GSUs in the Matters Arising. Nevertheless, based on our new evaluation, we found that there were indeed some cases where non-native distributions were not excluded despite trying to correct for that in our original dataset. In our published paper, we had cleaned our dataset to remove non-native distributions following the POWO database in May 2019, besides other data sources such as efloras (http://www.efloras.org/, accessed: May, 2019) (see Methods in ref. ^2^). Notably, the POWO has been under active development and the data in POWO are updated weekly. The POWO data for both native and non-native species distributions have grown very rapidly in the last few years. Specifically, the POWO database included 32,029 entries of non-native distributions in May 2019 when we cleaned our data in our published paper^2^, but included 49,526 entries by August 2023. Here, we updated our database by removing the newly-identified non-native distributions in POWO obtained in August 2023, and repeated our regionalization analyses. We found that our regionalization map remained largely unchanged (Figs. 1 and 2). Notably, since we focus on the whole angiosperm genera at global scale, it is very difficult for us to exclude all the non-native records, because the non-native records update continuously through time. However, our comparison here indicates that non-native distributions did not bias the results of our study^2^.

## Data coverage

We used >1100 data sources available by May 2019 for the compilation of genus distributions, as stated in the Method section of ref. ^2^ Currently, our database included ca. 384,000 entries, which are more than those contained in the POWO database. Our database compiles online databases, regional and national floras as well as published literatures and community studies, and has good coverage also on regions (e.g., China and Russia) with limited data in online databases such as the POWO database and the Global Biodiversity Information Facility (GBIF, https://www.gbif.org/). Although the POWO database is one of these data sources, the majority of our distribution data was not from POWO. Because of mismatches between the boundaries of our GSUs and the POWO map, a large proportion of the distribution data in POWO cannot be precisely adapted to our GSUs. In the published Supplementary Information of ref. ^2^ (Fig. S9), we addressed the issue of incomplete sampling (also see “Sensitivity analyses for the uncertainty in identifying floristic realms” in ref. ^2^). To further evaluate the potential influence of incomplete sampling on our results, we included the distribution data from the POWO database that are new to our database and can be precisely adapted to it (i.e., the geographic units used in POWO that are comparable to, or a subset of, the GSUs used in our study). We then repeated the regionalization analysis and showed that the regionalization remains largely unchanged, suggesting that the regionalization regime is robust to small variations in distribution data (see Figs. 1 and 2; also see Figs. 1 and 2 in ref. ^2^). The one border that shifts visibly is the border between the Saharao-Arabian and African realms in maps at 0 Ma and 10 Ma, but all the maps before 20 Ma remain unchanged. Notably, new distribution data are accumulated continuously with the development of many databases including the POWO, which might improve the issue of incomplete sampling, although it likely cannot be totally solved.

## Methods

The original distributional data were presented in Supplementary Data 2 of ref. ^2^ Distributional data from POWO were obtained using the R package rWCVP 1.2.4^3^. To determine the origin of the distribution of each genus in each geographic unit (native vs. non-native), we matched the geographic units of our study with those of POWO. Notably, the species distribution maps in the POWO follow the World Geographical Scheme for Recording Plant Distributions (WGSRPD level 3, https://www.tdwg.org/standards/wgsrpd/). The boundaries of WGSRPD differ from the geographical standard units (GSU) used in our study. This inconsistency between the boundaries of the POWO map and our GSUs leads to difficulties in the evaluation of non-native distributions. To facilitate the comparison, we overlaid our GSU map with WGSRPD (level 3) in ArcGIS 10.0 to match our GSUs with the geographic units in the POWO map. A GSU is assigned to a WGSRPD unit if >25% of its area overlaps with a WGSRPD unit. There are 401 GSUs that matched to one WGSRPD unit (i.e., the geographical unit used in POWO) and 19 GSUs were covered by two WGSRPD units. Then we calculated the number of genera containing non-native distributions and the total number of non-native occurrence records in our database. For comparison, we further changed the threshold for matching our GSUs with WGSRPD units to 50% and repeated the analysis. The results based on these two thresholds were consistent.

To evaluate whether non-native distributions and incomplete sampling may influence our results, we updated our database by removing the newly identified non-native data and including the distributions from POWO that are new and can be precisely adapted to our database. Using this updated database, we repeated our regionalization analyses (see Methods of ref. ^2^).

### Reporting summary

Further information on research design is available in the Nature Portfolio Reporting Summary linked to this article.

### Supplementary information

Reporting Summary

## Data Availability

We have updated the distribution data by removing non-native data as listed in the POWO and adding distribution data from the POWO that are new to our database. We have also updated the regionalization map on the website provided in the data availability section of ref. ^2^ (https://en.geodata.pku.edu.cn/index.php?c=content&a=list&catid=199). The original dataset can still be obtained from Supplementary Data 2 of ref. ^2^ All codes needed to evaluate the conclusions in the paper can be found at https://github.com/yunpengliu1994/regionalization (10.5281/zenodo.7758185).
