# Supplementary material for: Reply to: Reassessing data quality underlying the recently updated floristic map of the world
Source: Nat Commun. 2024 May 2;15:3673. doi: 10.1038/s41467-024-47544-6 (PMC11066100; doi:10.1038/s41467-024-47544-6)
Supplement: Supplementary file 1 — Reporting Summary [file 41467_2024_47544_MOESM1_ESM.pdf]

nature portfolio

Corresponding author(s): Carsten Rahbek; Zhiheng Wang

Last updated by author(s): Dec 5, 2023

Reporting Summary

Nature Portfolio wishes to improve the reproducibility of the work that we publish. This form provides structure for consistency and transparency in reporting. For further information on Nature Portfolio policies, see our Editorial Policies and the Editorial Policy Checklist.

Statistics

For all statistical analyses, confirm that the following items are present in the figure legend, table legend, main text, or Methods section.

n/a	Confirmed	
x		The exact sample size (*n*) for each experimental group/condition, given as a discrete number and unit of measurement
x		A statement on whether measurements were taken from distinct samples or whether the same sample was measured repeatedly
x		The statistical test(s) used AND whether they are one- or two-sided<br>*Only common tests should be described solely by name; describe more complex techniques in the Methods section.*
x		A description of all covariates tested
x		A description of any assumptions or corrections, such as tests of normality and adjustment for multiple comparisons
x		A full description of the statistical parameters including central tendency (e.g. means) or other basic estimates (e.g. regression coefficient) AND variation (e.g. standard deviation) or associated estimates of uncertainty (e.g. confidence intervals)
x		For null hypothesis testing, the test statistic (e.g. *F*, *t*, *r*) with confidence intervals, effect sizes, degrees of freedom and *P* value noted<br>*Give P values as exact values whenever suitable.*
x		For Bayesian analysis, information on the choice of priors and Markov chain Monte Carlo settings
x		For hierarchical and complex designs, identification of the appropriate level for tests and full reporting of outcomes
x		Estimates of effect sizes (e.g. Cohen's *d*, Pearson's *r*), indicating how they were calculated

Our web collection on statistics for biologists contains articles on many of the points above.

Software and code

Policy information about availability of computer code

Data collection	R package rWCVP 1.2.4
Data analysis	ArcGIS 10.0

For manuscripts utilizing custom algorithms or software that are central to the research but not yet described in published literature, software must be made available to editors and reviewers. We strongly encourage code deposition in a community repository (e.g. GitHub). See the Nature Portfolio guidelines for submitting code & software for further information.

Data

Policy information about availability of data

All manuscripts must include a data availability statement. This statement should provide the following information, where applicable:

- Accession codes, unique identifiers, or web links for publicly available datasets
- A description of any restrictions on data availability
- For clinical datasets or third party data, please ensure that the statement adheres to our policy

We have updated the distribution data by removing non-native data as listed in POWO and adding distribution data from POWO that are new to our database. We have also updated the regionalization map on the website provided in the data availability section of Liu et al. (https://en.geodata.pku.edu.cn/index.php?c=content&a=list&catid=199 (User: flowertree; Password: flowertree). The original dataset can still be obtained from the Supplementary Data 2 of Liu et al. (2023) Nat. Comm..

Research involving human participants, their data, or biological material

Policy information about studies with human participants or human data. See also policy information about sex, gender (identity/presentation), and sexual orientation and race, ethnicity and racism.

Reporting on sex and gender	No sex or gender involved
Reporting on race, ethnicity, or other socially relevant groupings	No race, ethnicity or other socially relevant groupings involved
Population characteristics	Not applicable
Recruitment	Not applicable
Ethics oversight	Not applicable

Note that full information on the approval of the study protocol must also be provided in the manuscript.

Field-specific reporting

Please select the one below that is the best fit for your research. If you are not sure, read the appropriate sections before making your selection.

☐ Life sciences ☐ Behavioural & social sciences ☒ Ecological, evolutionary & environmental sciences

For a reference copy of the document with all sections, see nature.com/documents/nr-reporting-summary-flat.pdf

Ecological, evolutionary & environmental sciences study design

All studies must disclose on these points even when the disclosure is negative.

Study description	This is a reply to Reassessing data quality underlying the recently updated floristic map of the world
Research sample	The original distributional data were presented in Supplementary Data 2 of Liu et al. (2023) Nat. Comm. Distributional data from POWO were obtained using the R package rWCVP 1.2.4.
Sampling strategy	All data are used
Data collection	All data for the present analyses was retrieved from public and from published datasets as listed in Method section of the reply. Yunpeng Liu compiled the distributional dataset.
Timing and spatial scale	POWO data are collected at Aug 7th, 2023 at global scale
Data exclusions	No data were excluded
Reproducibility	Not applicable
Randomization	Not applicable
Blinding	This study does not involve trials or similar experiments. Therefore, blinding is not relevant to this study.

Did the study involve field work? ☐ Yes ☒ No

Reporting for specific materials, systems and methods

We require information from authors about some types of materials, experimental systems and methods used in many studies. Here, indicate whether each material, system or method listed is relevant to your study. If you are not sure if a list item applies to your research, read the appropriate section before selecting a response.

Materials & experimental systems

n/a	Involved in the study
☒	☐ Antibodies
☒	☐ Eukaryotic cell lines
☒	☐ Palaeontology and archaeology
☒	☐ Animals and other organisms
☒	☐ Clinical data
☒	☐ Dual use research of concern
☒	☐ Plants

Methods

n/a	Involved in the study
☒	☐ ChIP-seq
☒	☐ Flow cytometry
☒	☐ MRI-based neuroimaging

Plants

Seed stocks	Not applicable
Novel plant genotypes	Not applicable
Authentication	Not applicable
